# Levels of insecticide resistance to deltamethrin, malathion, and temephos, and associated mechanisms in *Aedes aegypti* mosquitoes from the Guadeloupe and Saint Martin islands (French West Indies)

**DOI:** 10.1186/s40249-017-0254-x

**Published:** 2017-02-10

**Authors:** Daniella Goindin, Christelle Delannay, Andric Gelasse, Cédric Ramdini, Thierry Gaude, Frédéric Faucon, Jean-Philippe David, Joël Gustave, Anubis Vega-Rua, Florence Fouque

**Affiliations:** 1 Laboratory of Medical Entomology, Unit Environment and Health, Pasteur Institute of Guadeloupe, 97183 Les Abymes, Guadeloupe; 2 Vector control Service, Regional Health Agency, Dothémare, Les Abymes, Guadeloupe; 3 Alpine Ecology Laboratory (LECA), CNRS, UMR 5553, 2233 rue de la piscine BP53, 38041 Grenoble, Cedex 9 France; 4 University of Grenoble Alpes, Grenoble, France; 5 Environmental and Systems Biology (BEeSy), University of Grenoble Alpes, Grenoble, France; 6 Vector Environment and Society Unit, Special Programme for Research and Training in Tropical Diseases (TDR), World Health Organization, 20, Avenue Appia, CH-1211 Geneva 27, Switzerland

**Keywords:** *Aedes aegypti*, Mosquitoes, Insecticide resistance, Deltamethrin, Malathion, Temephos, Guadeloupe, Saint Martin

## Abstract

**Background:**

In the Guadeloupe and Saint Martin islands, *Aedes aegypti* mosquitoes are the only recognized vectors of dengue, chikungunya, and Zika viruses. For around 40 years, malathion was used as a mosquito adulticide and temephos as a larvicide. Since the European Union banned the use of these two insecticide molecules in the first decade of the 21st century, deltamethrin and *Bacillus thuringiensis* var. *israelensis* are the remaining adulticide and larvicide, respectively, used in Guadeloupe. In order to improve the management of vector control activities in Guadeloupe and Saint Martin, we investigated *Ae. aegypti* resistance to and mechanisms associated with deltamethrin, malathion, and temephos.

**Methods:**

*Ae. aegypti* mosquitoes were collected from six different localities of Guadeloupe and Saint Martin. Larvae were used for malathion and temephos bioassays, and adult mosquitoes for deltamethrin bioassays, following World Health Organization recommendations. Knockdown resistance (*Kdr*) genotyping for V1016I and F1534C mutations, and expression levels of eight enzymes involved in detoxification mechanisms were examined in comparison with the susceptible reference Bora Bora strain.

**Results:**

Resistance ratios (RR_50_) calculated for *Ae. aegypti* larvae showed high resistance levels to temephos (from 8.9 to 33.1-fold) and low resistance levels to malathion (from 1.7 to 4.4-fold). Adult females displayed moderate resistance levels to deltamethrin regarding the time necessary to affect 50% of individuals, varying from 8.0 to 28.1-fold. Molecular investigations on adult mosquitoes showed high resistant allele frequencies for V1016I and F1534C (from 85 to 96% and from 90 to 98%, respectively), as well as an overexpression of the glutathione S-transferase gene, *GSTe2*, the carboxylesterase *CCEae3a*, and the cytochrome genes *014614*, *CYP6BB*2, *CYP6M11*, and *CYP9J23*.

**Conclusions:**

*Ae. aegypti* populations from Guadeloupe and Saint Martin exhibit multiple resistance to organophosphates (temephos and malathion), and pyrethroids (deltamethrin). The mechanisms associated with these resistance patterns show strong frequencies of F1534C and V1016I *Kdr* mutations, and an over-expression of *CCEae3a*, *GSTe2*, and four cytochrome P450 genes (*014614*, *CYP9J23*, *CYP6M11*, *CYP6BB2*). These results will form the baseline for a deeper understanding of the insecticide resistance levels and associated mechanisms of *Ae. aegypti* populations and will be used to improve vector control strategies in Guadeloupe and Saint Martin.

**Electronic supplementary material:**

The online version of this article (doi:10.1186/s40249-017-0254-x) contains supplementary material, which is available to authorized users.

## Multilingual abstracts

Please see Additional file 1 for translations of the abstract into the six official working languages of the United Nations.

## Background

Recently, from December 2013 to January 2015, the chikungunya virus (CHIKV) intensely hit the Guadeloupe islands of the French West Indies, which resulted in a severe outbreak in which an estimated 40% (160,000 people) of the population became infected [1, 2]. Most of the Latin American countries and Caribbean islands were still reporting CHIKV outbreaks when a new threat was reported from Brazil—the arrival of the Zika virus (ZIKV) [3]. This arbovirus, new for the American region, was first considered as a mild disease until the discovery of Guillain-Barré and microcephaly syndromes associated with ZIKV infections [4]. Since then, the number of severe cases has drastically increased in Brazil and in most of the Latin American countries and Caribbean islands where ZIKV has been reported, and the first severe neurological cases associated with the ZIKV in the French West Indies were reported from Martinique in early 2016 [5]. In the French West Indies, both CHIKV and ZIKV are transmitted by the mosquito species *Aedes aegypti*, the vector of the dengue viruses that cause epidemics in the region every 2 to 3 years [6].

Since no specific treatment and/or vaccines are commonly available against CHIKV and ZIKV, and since the dengue vaccine has been deployed only very recently in a small number of experimental areas [7], the control of vectors and personal protection against mosquito bites remain the only available tools for preventing and controlling these emerging arboviral diseases [8, 9].

Historically, vector control activities in Guadeloupe have been carried out by a Vector Control Agency from the French Ministry of Health local delegation (Agence Régionale de Santé, ARS) and by ensuring larval reduction through the elimination of breeding sites and larvicide treatments, as well as the spraying of adulticide outdoors and indoors [10]. Biological larval control has also been tested using larvivorous fish in bigger tanks (J. Gustave, personal communication). The insecticides commonly used in the past have been temephos as a larvicide, and malathion and deltamethrin as adulticides [11]. However, since 2009, temephos has been withdrawn from the list of vector control insecticides, following European Union recommendations, and has been replaced by biological products using *Bacillus thuringiensis* var. *israelensis* (Bti) [12]. In 2010, the adulticide malathion was also withdrawn, consequently, the vector control activities in Guadeloupe were reorganized and now comprise routine elimination of breeding sites, the use of Bti as a larvicide, and the use of deltamethrin as an indoor adulticide.

In the French overseas departments of Guadeloupe, French Guiana, and Martinique, the surveillance of arbovirus epidemics is organized around a specific system called the *dengue epidemics surveillance, alert and management program* (PSAGE). Surveillance and control measures increase if necessary, according to the epidemiological situation, which is monitored all year round through a network of physicians in each territory who report weekly numbers of clinical cases for dengue, CHIKV, and now ZIKV. The PSAGE system proposes five operational situations [http://opac.invs.sante.fr/doc_num.php?explnum_id=3517]: phase 1) inter-epidemic situation with sporadic transmission; phase 2) some clusters of cases, seasonal increase, outdoor spraying of deltamethrin in and around clusters; phase 3) pre-epidemic alert with a significant increase in the number of clinical cases, outdoor spraying of deltamethrin in selected areas, reinforcement of entomological surveillance; phase 4) outbreak alert, intensification of outdoor spraying of deltamethrin in all places, communicating messages adapted to the epidemic context (including personal protection and reinforcement of personal elimination of breeding sites) to populations; and the final phase 5) end of the epidemic and evaluation of the lessons learned.

The large deployment of insecticides to control mosquito vectors has allowed the development of resistance worldwide [13]. For the Guadeloupe islands, very few studies have been done on the resistance levels of the mosquito *Ae. aegypti* [10], and resistance mechanisms have not been investigated. The resistance of mosquitoes to chemical insecticides can be attributed to two main mechanisms [14]. The first mechanism is the modification of the molecular site targeted by the chemical, and the second is an increased metabolism of the chemical through mutations and/or over-expression of detoxifying enzymes.

First report of *Ae. aegypti* insecticide resistance in Guadeloupe and Saint Martin are almost 20 years old, and resistance levels have probably significantly evolved with the increasing insecticide dosages. Thus, an update of *Ae. aegypti* resistance levels is urgently needed to improve the management of vector control activities in the Guadeloupe and Saint-Martin islands. Therefore, in this study, we investigated insecticide resistance levels and their associated molecular mechanisms for six *Ae. aegypti* populations collected in Guadeloupe and Saint Martin islands between January 2014 and October 2015.

## Methods

### Mosquito collection and rearing

**Fig. 1:**
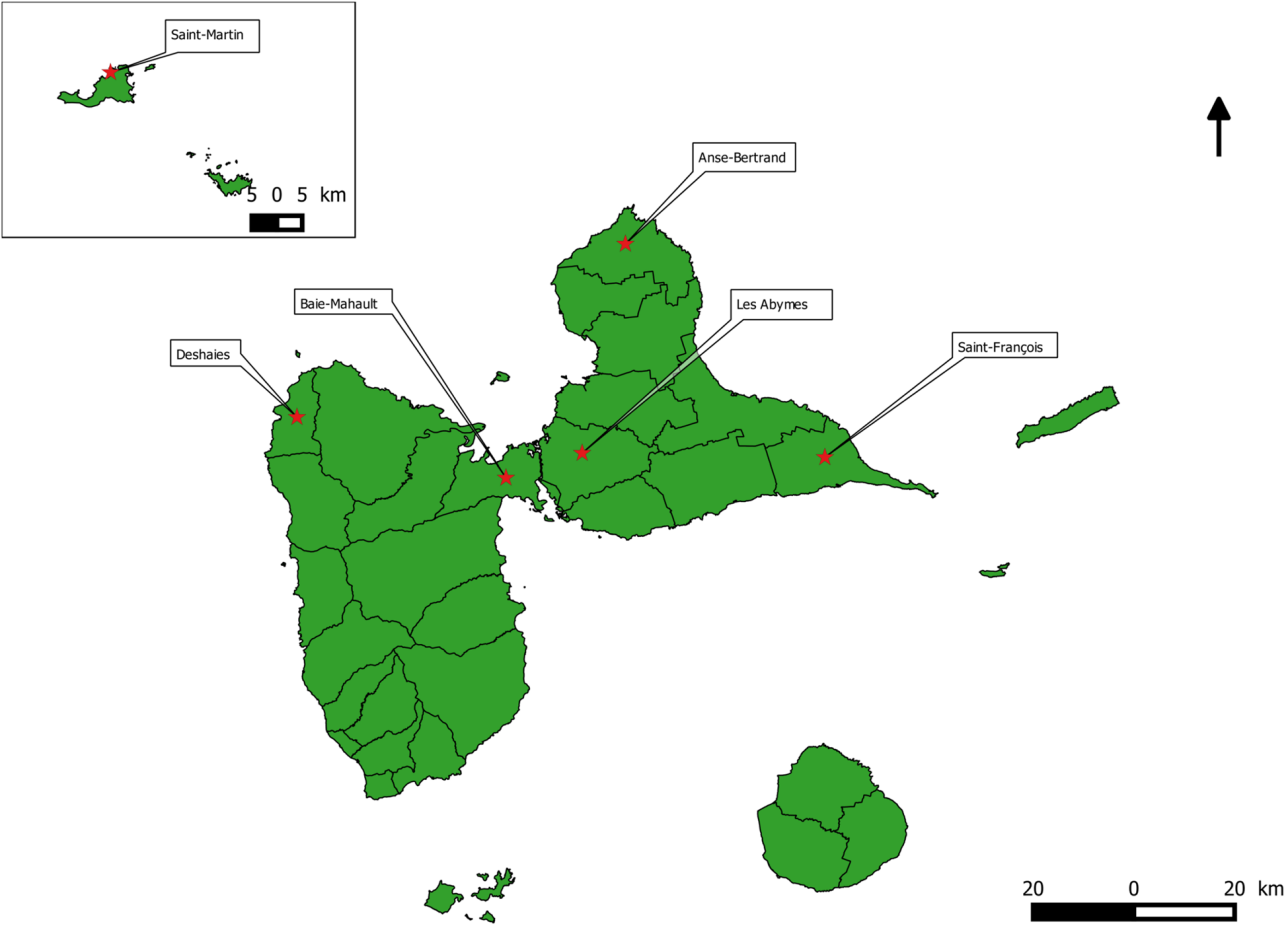
Geographical distribution of *Ae. aegypti* sampling sites in Guadeloupe and Saint Martin islands. In Guadeloupe, the sampling sites were located in Grande-Terre island (Anse-Bertrand, Saint-François, Les Abymes), and Basse-Terre island (Baie-Mahault and Deshaies)

*Ae. aegypti* mosquitoes were collected as larvae or pupae in urban, suburban, and rural areas from six different locations of Guadeloupe and Saint Martin (see Table 1 and Fig. 1). Mosquitoes were collected around private houses and in public areas, and were chosen randomly. Larvae and pupae were brought back to the insectarium facilities and reared in containers with around 150 to 200 mosquitoes per liter of dechlorinated tap water and supplemented with one yeast tablet at a constant temperature of 27° ± 1 °C, 80% of humidity, and a 12-h light/12-h dark cycle. Emerged adults were kept in cages and fed with a 10% sucrose solution. In order to produce the first generation (F1) of mosquitoes, the females were fed with fresh human blood using a Hemotek feeding system (Hemotek Ltd. Great Britain, United Kingdom). Human blood was chosen because the natural *Ae. aegypti* populations of Guadeloupe are highly anthropophilic and do not easily feed on other blood sources. Samples were taken from the investigators in the medical laboratory of the Pasteur Institute of Guadeloupe. The first laboratory generation(F1) mosquitoes were used for all experiments and molecular biology investigations, except for mosquitoes from Baie-Mahault and Deshaies for which second laboratory generation (F2) mosquitoes were used. Bora-Bora susceptible mosquitoes were provided by the Martinique mosquito control Agency at the egg stage and reared under the same conditions. The Bora-Bora strain is considered as a reference for insecticide susceptibility tests as previously used in Marcombe et al. 2009 [15]. All of the mosquito collections were done between January 2014 and October 2015 (see Table 1).

**Table 1:** *Ae. aegypti* mosquito populations used in the study

Mosquito population	Collection site	GPS coordinates	Breeding sites: type (number)	Date of collection	Conducted tests
ABY	Les Abymes	16°14'09.7"N 61°30'20.9"W	tires (multiple)	14 Jan 2014	Larval test
ABY	Les Abymes	16°14'09.7"N 61°30'20.9"W	tires (multiple); casks (4); buckets (2)	23 Jan 2015	Adult test
ABY	Les Abymes	16°17'57.5"N 61°29'25.6"W	casks (1)	30 Jan 2015	Adult test
ABY	Les Abymes	16°16'03.5"N 61°30'09.5"W	casks (2); bucket (1)	30 Jan 2015	Adult test
SF	Saint-François	16°15'N 61°16'W	aquatic plants (2); plant cutting (1)	16 Jan 2014	Larval test
SF	Saint-François	16°16'N 61°16'W	plant cutting (2)	16 Jan 2014	Larval test
SF	Saint-François	16°17'N 61°17'W	buckets (9); casks (3); small wastes (4); pot dish (1)	24 Sep 2015	Adult test
BM	Baie-Mahault	16°14'00.2"N 61°36'36.8"W	plant cutting (1); bucket (1)	22 Jan 2014	Larval test
BM	Baie-Mahault	16°15'30.9"N 61° 35'14.6"W	watering can (1)	22 Jan 2014	Larval test
BM	Baie-Mahault	16°15'09.0"N 61°35'54.9"W	plant cutting (1); bucket (1)	13 Feb 2015	Adult test
BM	Baie-Mahault	16°14'22.8"N 61°36'03.7"W	Cask (1)	13 Feb 2015	Adult test
SXM East	Saint Martin East French part	18°03'N 63°01'W	abandoned boat (1); abandoned jacuzzi (1); tires (multiple); casks (2); discarded small containers (1)	04–05 Feb 2014	Larval test
SXM West	Saint-Martin West French part	18°03'N 63°06'W	tires (2); casks (3); small waste (1); plant cutting (1); pot dish (1); buckets (3)	04–05 Feb 2014	Larval test
SXM	Saint-Martin	18°04'N 63°03'W	abandoned boat (1); tires (multiple); abandoned jet ski (1); discarded small containers (1)	25–26 Nov 2014	Adult test
AB	Anse-Bertrand	16°26'N 61°28'W	pot dish (2); flower vase (1); casks (4); watering can (1); bucket (2); tires (5)	07 Oct 2015	Larval and Adult test
DH	Deshaies	16°18'N 61°47'W	casks (2); bucket (1); flower pot (1); watering can (1); tires (multiple)	06 Oct 2015	Larval and Adult test

*ABY* Les Abymes, *SF* Saint-François, *BM* Baie-Mahault, *SXM* Saint Martin, *AB* Anse-Bertrand, *DH* Deshaies

### Larval bioassays

Larval bioassays were performed following WHO recommendations [16]. The late third- and early fourth-instar larvae were used for each mosquito population. Four replicates per concentration and five concentrations were tested with 25 larvae per replicate and per concentration. The insecticide alcohol dilutions were provided by the WHO Malaysia manufacturer (Vector Control Research Unit, School of Biological Sciences, Universiti Sains Malaysia). The insecticide solutions received were temephos (156.25 μg/ml and 31.25 μg/ml) and malathion (156.25 μg/ml and 31.25 μg/ml). To test the mosquito field samples and the Bora- Bora strain, concentrations were adjusted to include different percentages of survival/mortality, varying between 0 and 100%. Larvae were not fed during the 24 h of insecticide exposure. The concentrations tested for field samples were 1.5, 0.3, 0.15, 0.05, 0.015 μg/ml, and 1, 0.4, 0.2, 0.1, 0.04 μg/ml, for temephos and malathion, respectively (from insecticide solutions at 156.25 μg/ml); and 0.009, 0.0075, 0.006, 0.004, 0.0025 μg/ml (temephos), and 0.1, 0.075, 0.05, 0.04, 0.025 μg/ml (malathion) for the reference Bora Bora strain (from insecticide solutions at 31.25 μg/ml). The results were analyzed with XLSTAT-Biomed software (XLSTAT 2015 France) to determine the lethal concentration for 50% (LC_50_) and 95% (LC_95_) of the populations. Resistance ratios (RR_50_ and RR_95_) were calculated using LC_50_ and LC_95_ rates from *Ae. aegypti*-field-sampled populations compared with the LC_50_ and LC_95_ rates of the susceptible Bora Bora strain. The resistance levels were ranked into three categories: low resistance (RR_50_ < 5), medium or moderate resistance (5 ≤ RR_50_ ≤ 10), and high resistance (RR_50_ > 10) [17, 18].

### Adult bioassays

Adult bioassays were performed following WHO recommendations adapted for this study [19, 20]. Deltamethrin-impregnated papers were used at three different concentrations: 0.05, 0.06, and 0.08%. For each mosquito population and each concentration, four replicates and two negative controls of 25 females each were tested. The 0.05% deltamethrin-impregnated papers were ordered from the WHO Malaysia provider, and the 0.06 and 0.08% deltamethrin papers were impregnated in the laboratory using the deltamethrin, PESTANAL® (Sigma-Aldrich, Inc. Missouri, USA) in a solution of two-thirds acetone (Sigma-Aldrich, Inc.) and one-third silicone (VWR® Pennsylvanie, USA) with Whatman® Grade 1 Qualitative Filtration Paper (Sigma-Aldrich, Inc. Missouri, USA). The impregnation was done following WHO procedures [19] using 2 ml of insecticide solution to impregnate one 12 × 15 cm paper that was dried for one night and stored at 4 °C. Adult mosquitoes were exposed by tarsal contact for 1 h to the 0.06 and 0.08% deltamethrin-impregnated papers. The knockdown (KD) (after 1 h of exposure) and mortality rates (after 24 h) were determined. A kinetic of KD rate during 2 h (by accounting the number of KD mosquitoes every 5 min) was also conducted for each population using 0.05% impregnated papers to estimate the KDT_50_ (Time necessary to affect 50% of mosquitoes) using XLSTAT software (dose-effect option). The KRR_50_ is the ratio between KDT_50_ of mosquitoes from the field and KDT_50_ of mosquitoes from the Bora Bora strain. KRR_50_ was scaled as follows: KRR_50_<1 = susceptible, 1≤KRR_50_<10 = low resistance, 10≤KRR_50_≤30 = moderate resistance, 30<KRR_50_<100 = high resistance, and KRR_50_≥100 = very high resistance [21].

### Knockdown resistance (*Kdr*) genotyping

Total DNA of 24 single female mosquitoes per *Ae. aegypti* population was extracted using the QIAamp® DNA Mini Kit (Qiagen, Redwood city, CA, USA), following the manufacturer’s instructions. The region of the gene encoding sodium channel was amplified using the primers summarized in Table 2. A guanine-adenine transition in the first position of 1016 encodes a valine/isoleucine replacement (V1016I), while a thymine-guanine transition in the second position of 1534 encodes a phenylalanine/cysteine replacement (F1534C) [22–24].

**Table 2:** Primers for *Kdr* genotyping, according to [24, 45]

*Kdr* mutation	Primer name	Primer sequence (5' – 3')
V1016I	Val1016-f	GCGGGCAGGGCGGCGGGGGCGGGGCCACAAATTGTTTCCCACCCGCACCGG
	Iso1016-f	GCGGGCACAAATTGTTTCCCACCCGCACTGA
	Iso1011-r	GGATGAACCSAAATTGGACAAAAGC
F1534C	C1534-f	GCGGGCAGGGCGGCGGGGGCGGGGCCTCTACTTTGTGTTCTTCATCATGTG
	F1534-f	GCGGGCTCTACTTTGTGTTCTTCATCATATT
	CP-r	TCTGCTCGTTGAAGTTGTCGAT

The allele-specific real-time quantitative polymerase chain reaction (PCR) using SYBR® Green dye (Applied Biosystems®, Californie, USA) was done on an Applied Biosystems® 7500 thermal cycler (Californie, USA). The amplification consisted of a 95 °C 3 min holding stage, 40 cycles at 95 °C for 15 s, a 60 °C 31 s cycling stage, and a melt curve stage. For the PCR, a 15 μl solution comprising 7.5 μl of SYBR® Green PCR Master Mix, 0.4 μl of 10 μM reverse primer, 0.2 μl of 10 μM of both forward primers (C1534-f and F1534-f, see Table 2), 3.7 μl of H_2_O, and 3 μl of genomic DNA was made.

A Ile1016/Ile1016 homozygote (resistant) has a single peak at 76 °C; a Val1016/Val1016 homozygote (susceptible) has double peaks at 79 °C and 83 °C; and a Val1016/Ile1016 heterozygote has triple peaks at 76 °C, 79 °C, and 83 °C. A Cys1534/Cys1534 homozygote (resistant) has a single peak at 82 °C; a Phe1534/Phe1534 (susceptible) homozygote has a single peak at 78 °C; and a Phe1534/Cys1534 heterozygote has double peaks at 78 °C and 82 °C. Lastly, the distribution of the three genotypes for both mutations and allele frequencies were calculated.

### Gene expression study

RNA was extracted from three pools of 25 female mosquitoes each using the TRIzol®/chloroform (Invitrogen, Carlsbad, CA, USA) method, and cDNA was synthetized with SuperScript VILO Master Mix (Invitrogen) after a DNAse I treatment with DNAse I Amplification Grade (Invitrogen). A 15 ng/μl of cDNA was used for relative quantification PCR. For PCR, a 15 μl solution comprising 7.5 μl of SYBR® Green PCR Master Mix, 0.45 μl of 10 μM of each primer, 3.6 μl of H_2_O, and 3 μl of cDNA was made. The thermocycling conditions were the same as for *Kdr* genotyping. The differential expressions of eight candidate genes (*CCEae3a*, *CCEae6a*, *014614*, *CYP6BB2*, *CYP6M11*, *CYP9J23*, *CYP9J28*, and *GSTe2*; All primer sequences were designed by “Pollution, Environment, Ecological toxicology, Ecological remediation unit of Alpine Ecological Laboratory of Grenoble, summarized in Additional file 2) for the studied six populations were calculated using the ∆∆Ct method, taking into account PCR efficiency (see Table 3). Results show genes relative quantification for field mosquito populations compared to Bora Bora strain and using both RpS7 and RpL8 housekeeping genes (DataAssist™ v3.01 software).

**Table 3:** The 12 genes used in relative quantitative PCR [59]

Enzyme type	Accession number	Name (VectorBase)	Name in this study
Carboxyl/cholinesterase alpha esterase	AAEL005112	CCEae3a	CCEae3a
Carboxyl/cholinesterase alpha esterase	AAEL005122	CCEae6a	CCEae6a
Cytochrome P450	AAEL014614	ND	014614
Cytochrome P450	AAEL014893	CYP6BB2	CYP6BB2
Cytochrome P450	AAEL009127	CYP6M11	CYP6M11
Cytochrome P450	AAEL014615	CYP9J23	CYP9J23
Cytochrome P450	AAEL014617	CYP9J28	CYP9J28
Cytochrome P450	AAEL007808	CYP4D39	007808
Glutathione transferase	AAEL007951	GSTe2	GSTe2
60S ribosomal protein L8	AAEL000987	RpL8	RpL8
40S ribosomal protein S7	AAEL009496	RpS7	RpS7
Chloride channel protein 2	AAEL005950	ND	005950

### Gene amplification

The same DNA that was used for *Kdr* PCR was also used for the gene amplification study. Relative quantitative PCR was performed for five of the eight genes, i.e. *CCEae3a*, *014614*, *CYP6M11*, *CYP9J23*, and *GSTe2*. The PCR was done as previously described in the Gene expression study section above. Results show genes relative quantification for field mosquito populations compared to Bora Bora strain and using both 007808 and 005950 housekeeping genes (DataAssist™ v3.01 software).

### Statistical analysis

Statistical analyses were performed with STATISTICA 8 software (Statsoft Inc., Oklahoma, USA) and XLSTAT 2015 software. STATISTICA was used to perform ANOVA Kruskal-Wallis tests (to compare adult KD/mortality rates regarding deltamethrin and amplification gene ratios); Kruskal-Wallis Z tests (to compare larval mortality rates regarding temephos and malathion, adult mortality rates regarding deltamethrin, and expression and amplification genes ratios); and Mann–Whitney U tests (to compare gene expression ratios of Bora Bora strain and each population). XLSTAT was used to perform log-probit logistical regression to investigate the dose-effect relation regarding insecticide larval tests data, as well as to estimate KDT values using adult kinetic data. XLSTAT was also used to perform Spearman’s rank correlation tests (between expression and amplification ratios) and the principal component analysis (PCA).

## Results

### Larval bioassays

*Ae. aegypti* larvae of mosquito populations from the Guadeloupe and Saint Martin islands are highly resistant to temephos (see Table 4), with RR_50_ ranging from 8.9 (7.75–10.13) for Saint Martin West to 33.1 (29.63–37.75) for Les Abymes. The RR_95_ varied from 11.4 (10.21–13.07) for Saint Martin West to 39.8 (31.43–54.50) for Anse-Bertrand. The mosquito populations from Les Abymes and Baie-Mahault had the greatest resistance ratios, suggesting that these are the most resistant to temephos, followed by populations from Saint-François and Saint Martin East with intermediate levels of resistance, while mosquitoes from Saint Martin West had the lowest resistance ratio and were thus the most susceptible to temephos.

**Table 4:** Resistance status of *Ae. aegypti* larvae from Guadeloupe and Saint Martin, compared to the reference Bora Bora strain, to malathion and temephos

Population	Temephos						Malathion					
	LC_50_ (mg/L)	LC_50_ 95% *CI*	RR_50_	LC_95_ (mg/L)	LC_95_ 95% *CI*	RR_95_	LC_50_ (mg/L)	LC_50_ 95% *CI*	RR_50_	LC_95_ (mg/L)	LC_95_ 95% *CI*	RR_95_
ABY	0.265	0.237–0.302	33.125	0.529	0.461–0.632	37.786	0.285	0.265–0.307	4.385	0.464	0.429–0.510	3.899
SF	0.123	0.111–0.136	15.375	0.233	0.211–0.262	16.643	0.168	0.155–0.183	2.585	0.278	0.253–0.314	2.336
BM	0.233	0.211–0.259	29.125	0.450	0.402–0.519	32.143	0.277	0.256–0.299	4.262	0.465	0.428–0.515	3.908
SXM (West)	0.071	0.062–0.081	8.875	0.160	0.143–0.183	11.429	0.203	0.187–0.221	3.123	0.354	0.324–0.395	2.975
SXM (East)	0.128	0.112–0.144	16.000	0.303	0.273–0.345	21.643	0.239	0.219–0.262	3.677	0.449	0.409–0.502	3.773
AB	0.132	0.115–0.152	16.500	0.557	0.440–0.763	39.786	0.111	0.101–0.122	1.708	0.260	0.227–0.313	2.185
DH	0.151	0.132–0.172	18.875	0.555	0.442–0.758	39.643	0.109	0.099–0.119	1.677	0.238	0.208–0.284	2.000
Bora Bora	0.008	0.008–0.009	1.000	0.014	0.012–0.017	1.000	0.065	0.061–0.068	1.000	0.119	0.108–0.135	1.000

*LC*_*50*_ lethal concentration for 50% of individuals, *RR*_*50*_ resistance ratio between field samples and the Bora Bora strain, *LC*_*95*_ lethal concentration to 95% of individuals, *RR*_*95*_ resistance ratio between field samples and Bora Bora strain, *ABY* Les Abymes, *SF* Saint-François, *BM* Baie-Mahault, *SXM* Saint-Martin, *AB* Anse-Bertrand, *DH* Deshaies

The differences in the levels of resistance observed between *Ae. aegypti* populations for temephos were tested for statistical significance using the Kruskal-Wallis Z test, which highlighted significantly higher resistance levels among mosquitoes from Les Abymes and Baie-Mahault, compared to Saint-François and Saint Martin (*P* < 0.001). In addition, significantly higher resistance levels were also observed in the three mosquito populations from Anse-Bertrand, Saint-François, and Deshaies, compared to Saint Martin West (*P* = 0.000 for Anse-Bertrand, *P* = 0.027 for Saint-François, and *P* = 0.000 for Deshaies). Finally, the mosquitoes from Saint Martin West had a significantly higher mortality rate than all mosquito populations from Guadeloupe.

For malathion, the results for all field mosquito populations show some resistance in comparison with the susceptible Bora Bora strain (see Table 4), with the RR_50_ ranging from 1.7 for Anse-Bertrand and Deshaies, to 4.4 for Les Abymes. The RR95 varied from 2.0 for Deshaies to 3.9 for Les Abymes and Baie-Mahault.

The differences in the levels of resistance between *Ae. aegypti* populations were tested for statistical significance using the Kruskal-Wallis Z test. The results show higher mortality rates suggesting lower resistance for the mosquito populations of Anse-Bertrand and Deshaies compared to all other field populations (*P* < 0.01) except for Saint-François (*P* = 0.325 for Anse-Bertrand and *P* = 0.227 for Deshaies). The mosquito population of Saint-François showed a higher mortality rate than those from Les Abymes (*P* = 0.001) and Baie-Mahault (*P* = 0.002), which suggests that these two latter populations have a higher resistance to malathion.

### Adult bioassays

The KD rates, mortality rates, and resistance ratios (KRR_50_) were estimated for adult mosquitoes and are summarized in Table 5. The KD rates after 1 h of exposure varied from 0.93 (Deshaies) to 1.0 (Saint Martin), and 0.96 (Anse-Bertrand) to 1.0 (Saint-François, Saint Martin, Baie-Mahault, and Deshaies), for the 0.06 and 0.08% concentrations, respectively. The differences between the KD rates were significant between the mosquito populations of Anse-Bertrand (0.96) and Les Abymes (0.99), which had the lowest KD rates suggesting higher resistance to the 0.08% deltamethrin concentration (ANOVA Kruskal-Wallis: *P* = 0.011). Meanwhile, Deshaies (0.93) and Anse-Bertrand (0.94) mosquito populations had the lowest KD rates for the 0.06% deltamethrin concentration (ANOVA Kruskal-Wallis: *P* = 0.000).

**Table 5:** Resistance status of *Ae. aegypti* females from Guadeloupe and Saint Martin to deltamethrin

Deltamethrin concentration/time of exposure	Assessed parameters	DH	AB	SF	SXM	ABY	BM
0.06%/1 h	KD rate	0.93	0.94	0.98	1	0.96	0.99
	Mortality rate	0.83	0.64	0.79	0.88	0.79	0.90
0.08%/1 h	KD rate	1	0.96	1	1	0.99	1
	Mortality rate	0.94	0.93	0.81	0.98	0.85	0.99
0.05%/2 h	KRR50 (ci 95%)	17.7 (16.7–20.5)	28.1 (24.2–34.9)	13.7 (13.0–14.4)	12.4 (12.0–12.8)	8.0 (7.8–8.3)	13.6 (13.1–14.2)

KD rate: rate of knockdown mosquitoes after one hour of exposition, KDT_50_: time (min) necessary to knock down 50% of mosquitoes

KDT_50_ were determined using papers impregnated with 0.05 g/100 ml deltamethrin following WHO insecticide testing recommendations, KRR_50_: ratio between field samples KDT_50_ and susceptible Bora Bora strain KDT_50_, *DH* Deshaies, *AB* Anse-Bertrand, *SF* Saint-François, *SXM* Saint-Martin, *ABY* Les Abymes, *BM* Baie-Mahault

The mortality rates after 24 h following a 1-h exposure to a 0.06% deltamethrin concentration were significantly different between mosquito populations from Anse-Bertrand (0.64) and Baie-Mahault (0.90) (Kruskal-Wallis Z test, *P* = 0.040). The mortality rates after 24 h following a 1-h exposure to a 0.08% deltamethrin concentration were significantly different, with mosquito populations from Saint-François and Les Abymes exhibiting the lowest mortality rates, thus suggesting higher resistance levels (ANOVA Kruskal-Wallis: *P* < 0.0001).

KRR50 values were variable, ranging from 8.0 (Les Abymes) to 28.1 (Anse-Bertrand), indicating a low to moderate resistance level for deltamethrin when compared to the susceptible Bora Bora strain. Two levels of resistance were detected in the six field mosquito populations. The mosquito population of Les Abymes had the lowest KRR50 (8.0), suggesting a low resistance level to deltamethrin. All others mosquito populations had a KRR_50_ of between 12 and 28, and are considered moderately resistant.

### *Kdr* genotyping

**Fig. 2:**
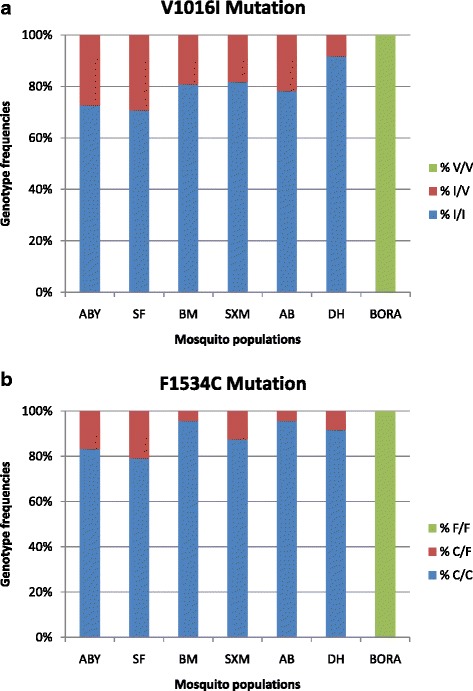
Histogram of genotype proportions for the six populations studied regarding V1016I (**a**) and F1534C (**b**) *Kdr* mutations. V/V: V1016I wild homozygote genotype; I/V: V1016I heterozygote genotype; I/I: V1016I mutant homozygote; F/F: F1534C wild homozygote genotype; F/C: F1534C heterozygote genotype; C/C: F1534C mutant homozygote; *ABY* Les Abymes, *SF* Saint-François, *BM* Baie-Mahault, *SXM* Saint Martin, *AB* Anse-Bertrand, *DH* Deshaies, *BORA* Bora Bora susceptible strain

Real-time PCR revealed high frequency of *Kdr* mutations V1016I and F1534C with high allelic frequencies (see Table 6). For the V1016I mutation, the frequency of the mutant and resistant allele (ƒ[I]) ranged from 0.85 (Saint-François) to 0.96 (Deshaies). For the F1534C mutation, the frequency of the mutant and resistant allele (ƒ[C]) ranged from 0.90 (Saint-François) to 0.98 (Anse-Bertrand and Baie-Mahault). No *Kdr* mutation was found in the susceptible Bora Bora strain. All mosquito populations exhibited more than 70% of the resistant homozygote genotype for both F1534C and V1016I mutations simultaneously (see Fig. 2). The F1534C *Kdr* mutation was more frequent in *Ae. aegypti* mosquitoes than the V1016I one because in five of the populations (Les Abymes, Baie-Mahault, Saint Martin, Anse-Bertrand, Deshaies), more than 80% of mosquitoes displayed the resistant homozygote genotype C/C of the F1534C mutation, while for the V1016I mutation, only three mosquito populations (Baie-Mahault, Saint-Martin, Deshaies) displayed the same frequency of resistant homozygote genotype I/I.

**Table 6:** Allele frequencies for the V1016I and F1534C *Kdr* mutations for each *Ae. aegypti* population

Population	Mutation V1016I ƒ[I]	Mutation V1016I ƒ[V]	Mutation F1534C ƒ[C]	Mutation F1534C ƒ[F]
ABY	0.86	0.14	0.92	0.08
SF	0.85	0.15	0.90	0.10
BM	0.90	0.10	0.98	0.02
SXM	0.91	0.09	0.94	0.06
AB	0.89	0.11	0.98	0.02
DH	0.96	0.04	0.96	0.04
Bora Bora	0.00	1.00	0.00	1.00

*ABY* Les Abymes, *SF* Saint-François, *BM* Baie-Mahault, *SXM* Saint Martin, *AB* Anse-Bertrand, *DH* Deshaies, *f[I]* allele frequency of the mutant allele I for *Kdr* mutation V1016I, *f[V]* allele frequency of the wild allele V for *Kdr* mutation V1016I, *f[C]* allele frequency of the mutant allele I for *Kdr* mutation F1534C, *f[F]* allele frequency of the wild allele V for *Kdr* mutation F1534C

### Detoxification enzyme levels

**Fig. 3:**
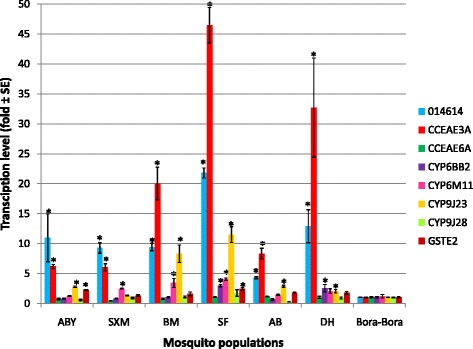
Adult transcription levels of five cytochrome P450 monooxygenases (014614, CYP6BB2, CYP6M11, CYP9J23, CYP9J28), two carboxyl/cholinesterases (CCEae3a, CCEae6a), and one glutathione S-transferase (GSTe2) for the six *Ae. aegypti* populations of Guadeloupe and Saint Martin, as compared to the susceptible Bora Bora strain. The transcription ratios obtained from real-time quantitative PCR were normalized with the two housekeeping genes RpL8 and RpS7 and shown as mean value (±SE) for three independent biological replicates. Genes significantly over-transcribed with transcription ratio ≥2 and *P*-value <0.05) are indicated by *asterisks. ABY* Les Abymes, *SXM* Saint Martin, *BM* Baie-Mahault, *SF* Saint-François, *AB*: Anse-Bertrand, *DH* Deshaies

The transcription profiles of the eight candidate detoxification genes potentially involved in metabolic resistance to insecticides were compared for the adults of the six mosquito populations of Guadeloupe and the susceptible Bora Bora strain (see Fig. 3). Genes with transcription ratio ≥2 and *P*-value < 0.05 (according to the Mann–Whitney *U* test done for each mosquito population and the Bora Bora strain) were considered significantly over-transcribed.

The cytochrome P450 monooxygenase, *014614*, was overexpressed in the six mosquito populations compared to the susceptible Bora Bora strain, and ranged from 4.3 for Anse-Bertrand to 21.8 for Saint-François, with significant differences in expression ratios between these two locations (Kruskal-Wallis Z test: *P* = 0.011). The cytochrome P450 monooxygenase, *CYP6BB2*, was found to be overexpressed only in the Saint-François and Deshaies populations with similar ratios of 3.0 and 2.5, respectively (Mann–Whitney *U* test: *P* = 0.512). The cytochrome P450 monooxygenase, *CYP6M11*, was over-transcribed for mosquito populations of Saint Martin, Baie-Mahault, and Saint-François, with ratios of 2.5, 3.4, and 4.0, respectively, and similar transcription levels (ANOVA Kruskal-Wallis: *P* = 0.201). The cytochrome P450 monooxygenase, *CYP9J23*, was significantly overexpressed for all mosquito populations with the exception of Saint Martin, with ratios ranging from 2.0 (Deshaies) to 11.5 (Saint-François). The transcription levels were significantly higher for Saint-François than for Deshaies (Kruskal-Wallis Z test: *P* = 0.026).

The carboxyl/cholinesterase 3A (*CCEea3a*) was over-transcribed in the six mosquito populations, with expression ratios ranging from 6.02 for Saint Martin to 46.5 for Saint-François. Mosquito populations of Saint-François (46.5-fold), Deshaies (32.7-fold), and Baie-Mahault (20.0-fold) exhibited higher levels of over-transcription than the rest of the populations (ANOVA Kruskal-Wallis: *P* = 0.011).

The glutathione S-transferase 2 (*GSTe2*) was over-transcribed only in the mosquito populations of Les Abymes and Saint-François with similar ratios of 2.3 and 2.4, respectively (Mann–Whitney *U* test: *P* = 0.218).

*CCEae6a* and the cytochrome P450 monooxygenase, *CYP9J28*, were not found to be over-transcribed in any of the populations when compared to the susceptible Bora Bora strain.

### Gene amplification study

**Fig. 4:**
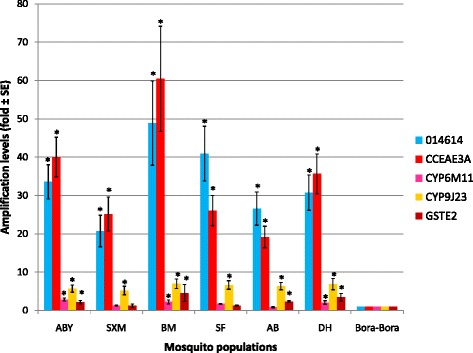
Adult gene amplification levels for three cytochrome P450 monooxygenases (014614, CYP6M11, CYP9J23), one carboxyl/cholinesterase (CCEae3a), and one glutathione S-transferase (GSTe2) for the six *Ae. aegypti* populations of Guadeloupe and Saint Martin compared to the susceptible Bora Bora strain. The gene amplification levels obtained from real-time quantitative PCR were normalized with both 007808 and 005950 housekeeping genes and shown as mean value (±SE) over 22 to 24 individual DNA. The genes significantly amplified (ratio ≥2) are indicated by *asterisks. ABY* Les Abymes, *SXM* Saint Martin, *BM* Baie-Mahault, *SF* Saint-François, *AB* Anse-Bertrand, *DH* Deshaies

The gene amplification profiles of five previously studied genes (*014614*, *CYP6M11*, *CYP9J23*, *CCEae3a*, and *GSTe2*) were compared between the susceptible Bora Bora strain and the six populations of Guadeloupe and Saint Martin (see Fig. 4).

The cytochrome P450 monooxygenase *014614* gene was amplified in all *Ae. aegypti* populations, with ratios ranging from 20.7 for Saint-Martin to 48.9 for Baie-Mahault. Significant differences in *014614* gene amplification were found between Saint Martin and both Baie-Mahault (Kruskal-Wallis Z test: *P* = 0.001) and Saint-François populations (Kruskal-Wallis Z test: *P* = 0.005). The cytochrome P450 monooxygenase, *CYP6M11*, was significantly amplified in the mosquito populations of Les Abymes, Baie-Mahault, and Deshaies, with ratios of 2.8, 2.2, and 2.0 respectively (ANOVA Kruskal-Wallis test: *P* = 0.036). The cytochrome P450 monooxygenase, *CYP9J23*, was amplified similarly in all *Ae. aegypti* populations with ratios of 5.2 to 7.0 (ANOVA Kruskal-Wallis test: *P* = 0.329). *CCEae3a* was amplified with ratios of >19 in all *Ae. aegypti* populations. Significant differences in amplification levels were observed between mosquito populations of Anse-Bertrand with a ratio of 19.2 and both Baie-Mahault and Les Abymes populations with ratios of 60.4 (Kruskal-Wallis Z test: *P* = 0.003) and 40.0 (Kruskal-Wallis Z test: *P* = 0.013), respectively. *GSTe2* was similarly amplified in mosquito populations from Baie-Mahault (4.5-fold), Deshaies (3.4-fold), Anse-Bertrand (2.3-fold), and Les Abymes (2.1-fold) (ANOVA Kruskal-Wallis test: *P* = 0.115).

### Correlation between overexpression and gene amplification of detoxification genes

Correlations between mean values of the expression and amplification ratios were investigated using the Spearman’s rank correlation test in XLSTAT software for each gene and all mosquito populations studied (see Additional file 3), however, no significant correlation was found. Nevertheless, the highest levels of association between gene amplification and expression ratios were found for the cytochrome P450 monooxygenases *CYP9J23* and *014614* (*r* = 0.543; *P* = 0.297), followed by *CCEae3a* (*r* = 0.200; *P* = 0.714). Finally, the cytochrome *CYP6M11* and *GSTe2* showed the weakest associations between gene amplification and expression levels (*r* = −0.143; *P* = 0.803).

### Association analysis between phenotypes, *Kdr* mutations, and detoxification enzymes regarding insecticide resistance

**Fig. 5:**
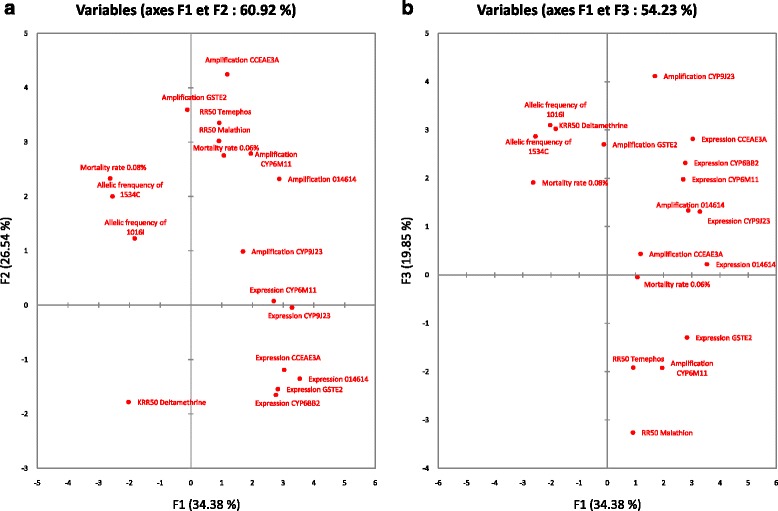
PCA for 18 variables including larval resistance levels regarding temephos and malathion, adult resistance levels regarding deltamethrin, adult transcription and amplification ratios of detoxification enzymes, and adult resistant *Kdr* allele frequencies, for the six mosquito populations tested. Information represented by axis 1-2 (**a**) and axis 1-3 (**b**) are shown

A PCA was performed for the six *Ae. aegypti* populations and 18 variables, including: (i) larval resistance levels regarding temephos and malathion, (ii) adult resistance levels regarding deltamethrin, (iii) adult transcription and amplification ratios of detoxification enzymes, and (iv) adult resistant *Kdr* allele frequencies (see Additional file 4). The PCA results are shown in Fig. 5.

The first three PCA axes allow to represent 76.77% of the starting information with 34.38, 26.54, and 19.85%, respectively. The expression of the *014614* gene was mainly represented on the first PCA axe (Cos^2^ = 0.811) and was found to be significantly correlated to those of *CYP6BB2* and *CCEae3a* genes (*r* = 0.870 and 0.853, respectively). The amplification ratio of *CCEae3a* was mainly represented on the second PCA axe (Cos^2^ = 0.901) and principally correlated with the *GSTe2* amplification ratio (*r* = 0.808) and RR50 for temephos (*r =* 0.769). The *CYP9J23* amplification ratio was mainly represented on the third axe (Cos^2^ = 0.633), and was found to be positively correlated with the *014614* and *GSTe2* amplification ratios (*r* = 0.712 and *r* = 0.680, respectively). Furthermore, the expression ratios of *CYP9J23* and *CYP6M11* genes were significantly correlated (*r* = 0.850). The allele frequencies of resistant *Kdr* 1534C and 1016I mutations were positively correlated (*r* = 0.611), but only the 1534C resistant mutant allele was correlated with deltamethrin resistance KRR50 (*r* = 0.611). Temephos resistance was significantly correlated with the amplification ratio of the *CYP6M11* gene (*r* = 0.849). Finally, the mortality rate after 24 h following a 1-h exposure to 0.08% deltamethrin was significantly and negatively correlated with the expression ratio of the *GSTe2* gene (*r* = −0.960).

## Discussion

The larval tests carried out on *Ae. aegypti* mosquito populations from Guadeloupe and Saint Martin showed that these mosquitoes have high resistance to temephos and low resistance to malathion. Tests done with adult mosquitoes showed moderate resistance levels to deltamethrin for KRR_50_s.

The chemical larvicide temephos was used for about 40 years, from the late 1960s to 2010, and resistance was reported as early as 1984 (Pasteur Institute archives). For adult vector control, malathion was used from the late 1960s to 2009, and deltamethrin from the early 1980s until the present day (Pasteur Institute archives). The mosquito populations of Guadeloupe and Saint Martin are reported to be resistant (weakly) to malathion for the first time in Guadeloupe (in this study), but are known to be resistant to deltamethrin since the late 1990s (Pasteur Institute and ARS archives). The long duration of use of temephos as a larvicide in Guadeloupe and Saint Martin, and the rapid increase of deltamethrin resistance may be explained by cross-resistance between these two molecules [25].

For temephos, the results of our study (i.e. RR_50_ between 8.9 and 33) are similar to those obtained for *Ae. aegypti* populations from the region, including in Martinique [26], Cuba, Venezuela, Costa Rica, Panama, Nicaragua, and Jamaica [27]. The detoxification enzymes commonly associated with organophosphates resistance are carboxylesterases (COEs) [28–30]. The gene expression study performed on adult mosquitoes revealed an overexpression for *CCEae3a*. Furthermore, the PCA showed a positive correlation between RR50 for temephos and *CCEae3a* amplification (*r* = 0.769). These results agree with previous studies demonstrating the implication of *CCEae3a* upregulation on larval temephos resistance [30]. In 1999, the higher resistance levels found for *Ae. aegypti* mosquito populations [10] were likely a consequence of the strong selective pressure exerted by the use of temephos as the main larvicide at the time especially due to its low price, efficacy and remanence. Since 2010, only Bti has been used in Guadeloupe as a larvicide, which explains the decrease in resistance levels and the lower resistance ratios found for temephos in this study.

For malathion, the resistance levels observed on larvae are low, with the RR50 ranging from 1.7 to 4.4 for all mosquito populations tested, when compared to the Bora Bora strain. Although malathion has been used for more than 40 years to control adult mosquitoes in Guadeloupe, the mosquito larvae did not develop a strong resistance to this molecule. This observation can be explained by the alternate use of malathion and deltamethrin since the late 1980s/early 1990s in Guadeloupe (Pasteur Institute archives), which may have delayed the emergence of higher resistance levels. Furthermore, the use of malathion in Guadeloupe has been intensified mostly during epidemic outbreaks. Another factor that may have mitigated the resistance level for malathion in mosquito populations is the possible dependency on a specific carboxylesterase mechanism with no cross-resistance with temephos. Our results on resistance levels to malathion are in agreement with recent studies conducted on *Ae. aegypti* larvae from Martinique [31], Cuba, Jamaica, Panama, Costa Rica [27], and adult mosquitoes from Venezuela [32].

Deltamethrin has been used since the late 1980s/early 1990s and is currently the only adulticide authorized in European countries, including the French overseas territories of Guadeloupe and Saint Martin. The observed knockdown deltamethrin resistance ratios (8≤KRR_50_≤28) suggest that the *Ae. aegypti* populations of Guadeloupe and Saint Martin are more resistant than those of Martinique [26]. On the contrary, when compared to French Guiana [33], *Ae. aegypti* populations from Guadeloupe were found to be less resistant in terms of KD (93–100% for Guadeloupe versus 27–37% for French Guiana) and mortality rates (64–90% for Guadeloupe versus 14–30% for French Guiana) obtained after exposure to 0.06% deltamethrin. Although our results contrast with those obtained by Dusfour and colleagues in 2015 [34] (in which lower KD and mortality rates, 37 and 42%, respectively, were obtained for the *Ae. aegypti* populations from Baie-Mahault), our study and the study of Dusfour and colleagues in 2015 show that the *Ae. aegypti* populations from Guadeloupe have lower resistance levels to deltamethrin than those from French Guiana.

It has been previously reported that COEs, cytochrome P450 monooxygenases, and GSTs are involved in pyrethroids resistance and metabolization [35–39]. The adult gene expression study performed on eight detoxification enzymes revealed a significant overexpression of the genes *GSTe2*, *CCEae3a*, and the cytochrome P450 monooxygenases *014614*, *CYP9J23*, *CYP6M11*, and *CYP6BB2*. The PCA revealed a significant and negative correlation between the GSTe2 expression ratio and the mortality rates obtained 24 h after a 1-h exposure to 0.08% deltamethrin. The overexpression of GSTe2 in *Ae. aegypti* resistant to DichloroDiphenylTrichloroethane (DDT) and permethrin had previously been reported in Thailand [40].

*Kdr* mutations are known to be strongly associated with organochloride and pyrethroid resistance for many mosquito species including *Ae. aegypti* [41, 42]. *Kdr* genotyping of the six mosquito populations studied revealed a highly resistant allele frequency of V1016I mutation ranging from 85 to 96%. These results are in agreement with those found for mosquito populations from Martinique and French Guiana, where frequencies ranged from 87 to 97% and from 65 to 92%, respectively [26, 33]. The mutant allele frequency of F1534C was also extremely high in populations from Guadeloupe, ranging from 90 to 98%. Similar frequencies of these mutations have been found in Mexico and Grand Cayman islands [43, 44]. A V1016I mutation has often been associated with permethrin resistance [45], while a F1534C mutation has been associated with a permethrin and DDT resistance [44]. The PCA revealed a positive correlation between the deltamethrin resistance ratio and the mutant allele frequency of F1534C (*r* = 0.611), but a low correlation with V1016I (*r* = 0.262). Similar observations have already been reported in 2013 for the mosquito populations from Martinique regarding the V1016I mutation [46].

In conclusion, our results highlight that deltamethrin resistance levels seem to be more linked to *Kdr* mutation 1534C and *GSTe2* at high concentrations, rather than other detoxification enzyme mechanisms. In terms of temephos and malathion, resistance levels were found to be more related to detoxification mechanisms involving esterase (*CCEae3a*) and cytochrome (*CYP6M11*) enzymes rather than *Kdr* mutations.

The levels of resistance of *Ae. aegypti* populations of the Guadeloupe and Saint Martin islands to the different types of insecticides are likely a consequence of vector control and agricultural activities. In Guadeloupe, lindane (ɣHCH) and chlordecone, two organochlorine pesticides, have been used in banana cultures to eliminate weevils in 1965–1974 and 1972–1993, respectively [47]. Furthermore, the principal active products of herbicides used in local sugar cane cultures are generally organophosphates and carbamates [http://e-phy.agriculture.gouv.fr/], while certain insecticides used in horticulture contain pyrethroids such as deltamethrin. Today, the most abundant pesticide in water in the French overseas departments is organochlorine [http://www.statistiques.developpement-durable.gouv.fr/lessentiel/ar/246/211/contamination-globale-eaux-souterraines-pesticides.html]. The long history of pesticide use in agriculture in Guadeloupe with the same classes of molecules applied for vector control could thus have contributed to the type of insecticide resistance in *Ae. aegypti* populations. This phenomenon has been commonly described for malaria vectors [48–50].

Finally, gene amplification is commonly associated with an overexpression of detoxification enzymes [13, 51, 52]. In our study, no significant correlation was found between expression and amplification ratios for any of the five genes tested, however, the strongest associations were recorded for *CYP9J23* and *014614* (*r* = 0.543).

## Conclusion

In the present study, the populations of *Ae. aegypti* mosquitoes—the main vector of dengue, CHIKV, and ZIKV—from the Guadeloupe and Saint Martin islands were found to have different resistance levels to several insecticides, with a high resistance to the former larvicide temephos, moderate resistance to the adulticide deltamethrin, and low resistance to the former adulticide malathion. The resistance levels are associated with strong frequencies of F1534C and V1016I *Kdr* mutations, as well as the over-transcription of *CCEae3a*, *GSTe2*, and four cytochromes P450 (*014614*, *CYP9J23*, *CYP6M11*, *CYP6BB2*) genes.

Mosquito resistance to deltamethrin is a serious challenge for vector control authorities since this product is the only adulticide authorized in the French overseas territories, in particular during epidemics. In addition, deltamethrin has also been used in agriculture for decades. In this context, there is an urgent need to carefully choose the molecules used for vector control and agricultural activities, as well as reduce their utilization in order to limit the chance of resistance. It is also urgent to identify alternative molecules that are effective and respectful of the environment that could be used in a vector control strategy based on a rotation and/or combination of insecticides (which leads to resistance development slowing down), as recommended by the WHO [53].

Our knowledge of the molecular basis of *Ae. aegypti* insecticide resistance has contributed to real progress in the past few years, but it is still not developed enough either to be used as a diagnostic tool for identifying resistance without the necessity to develop heavy testing procedures or understanding how the resistance mechanisms are developing. The present study elucidated that it could be interesting to: i) assess the activity of some families of detoxification enzymes using biochemical/enzymatic assays; ii) use synergists such as Triphenyl phosphate (COEs inhibitor), piperonyl butoxide (P450s inhibitor), or ethacrynic acid or diethyl maleate (GSTs inhibitor) in order to specify enzyme families and mechanisms involved in resistance [54]; iii) segregate tested mosquitoes and conduct quantitative PCR to understand induced gene expression and thus to specify the role of some genes individually; iv) add more detoxification genes in the analysis (i.e. *GSTe7*, *CYP6Z*) [26]; and v) investigate more *Kdr* mutations as I1011V which has already been associated to pyrethroid resistance in Cuba and Mexico [45], as well as I1011M and G923V in Brazil [55]. A better understanding of the resistant genes and their roles is needed in order to improve vector control strategies, mitigate insecticide resistance levels, and prevent and control the emergence and spread of *Ae. aegypti*-borne diseases.

## Additional files

Additional file 1: Abstracts in the six official working languages of the United Nations. (PDF 760 kb)
Additional file 2: Set of primers used for the expression and amplification of detoxification enzymes q-PCR. (DOCX 14 kb)
Additional file 3: Mean expression and amplification ratios used for Spearman’s rank correlation tests. (XLSX 11 kb)
Additional file 4: PCA matrix. (XLSX 11 kb)

## Abbreviations

ARS: Agence Régionale de Santé
Bti: *Bacillus thuringiensis* var. *israelensis*
CCEae: Carboxyl/cholinesterase
CHIKV: Chikungunya virus
COE: Carboxylesterase
F1: First laboratory generation mosquitoes
F2: Second laboratory generation mosquitoes
GST: Glutathione s-transferase
KD: Knockdown
KD: Knock-down
Kdr: Knockdown resistance
KDT: Knock-down time
LC: Lethal concentration
PCA: Principal component analysis
PCR: Polymerase chain reaction
PSAGE: Programme de Surveillance, d’Alerte et de Gestion des epidémies de dengue
RR: Resistance ratio
WHO: World Health Organization
ZIKV: Zika virus
