# Supplementary material for: Levels of insecticide resistance to deltamethrin, malathion, and temephos, and associated mechanisms in Aedes aegypti mosquitoes from the Guadeloupe and Saint Martin islands (French West Indies)
Source: Infect Dis Poverty. 2017 Feb 10;6:38. doi: 10.1186/s40249-017-0254-x (PMC5303256; doi:10.1186/s40249-017-0254-x)
Supplement: Additional file 1: — Abstracts in the six official working languages of the United Nations. (PDF 760 kb) [file 40249_2017_254_MOESM1_ESM.pdf]

Translation of the abstract into the six official working languages of the United Nations

## مستويات وآليات مقاومة بعوض الزاعجة المصرية للمبيدات الحشرية من نوع الدلتاميثرين والملاثيون وتيميفوس بجزر غوادولوب وسان مارتن (جزر الهند الغربية الفرنسية)

دانييلا غواندان، كريستال ديلاناي، أندريك جلاس، سيدريك رامديني، جويل غوستاف، أنوبيس فيغا رووا، فلورونس فوك.

**الملخص:**

**الخلفية:** يعد بعوض الزاعجة المصرية الناقل الوحيد المعترف به لفيروس حمى الضنك وشيكونغونيا والزيكا بجزر غوادولوب وسان مارتن. وقد استخدم الملاثيون كمبيد للباعوض البالغ والتيميفوس كمبيد لليرقات على مدى 40 سنة تقريبا. وبعد منع الإتحاد الأوروبي لكلا المبيدين في العقد الأول من القرن 21، أصبح كل من الدلتاميثرين والمبيد الحيوي *Bacillus thuringiensis var.israelensis* مبيدا البعوض البالغ واليرقات على التوالي المستخدمين بجزر غوادولوب. وبغرض تحسين إدارة نشاط مكافحة ناقلات الأمراض بجزر غوادولوب وسان مارتن، نقوم بتحري مقاومة بعوض الزاعجة المصرية وآلياتها لمبيد الدلتاميثرين والملاثيون وتيميفوس.

**المنهجية:** تم جمع بعوض الزاعجة المصرية من ستة مناطق مختلفة بجزر غوادولوب وسان مارتن. استخدمت اليرقات للاختبارات الأحيائية التي استعمل فيها الملاثيون والتيميفوس، والباعوض البالغ للإختبارات الأحيائية التي استعمل فيها الدلتاميثرين، تبعا لتوصيات المنظمة العالمية للصحة. تم فحص التنميط الوراثي لتطفير للمقاومة القصوى *(Kdr)* لـ V1016I وF1534C ، ومستويات التعبير لثمانية إنزيمات التي تدخل في آليات التخلص من السموم بالمقارنة مع سلالة البورا بورا.

**النتائج:** أوضحت نسب المقاومة ($RR_{50}$) لدى بعوض الزاعجة المصرية مستويات عالية من مقاومة مبيد التيميفوس (من 8.9 إلى 33.1 ضعف) ومستويات ضعيفة من مقاومة مبيد الملاثيون (من 1.7 إلى 4.4 أضعاف). وقد أظهرت الإناث البالغات مستويات معتدلة من المقاومة لمبيد الدلتاميثرين بالنظر للوقت اللازم للتأثير على 50 % من الأفراد، مستويات تتراوح بين 8.0- 28.1 ضعف. أوضح التحري الجزيئي للبعوض البالغ مستويات عالية من المقاومة للتردد الجيني لـ V1016I وF1534C (من 85% إلى 96% ومن 90% إلى 98% على التوالي)، إضافة إلى إفراط في التعبير لجين الإنزيم ناقل الجلوتاثيون، *GSTe2*، وإستيراز الكربوكسيل *CCEae3a*، وجينات السيتوكروم *014614*، *CYP6BB2*،*CYP9J23* *CYP6M11* .

**الاستنتاج:** يظهر بعوض الزاعجة المصرية بجزر غوادولوب وسان مارتن مقاومة متعددة للمركبات العضوية الفوسفاتية (الملاثيون وتيميفوس) والبيروثرويدات (الدلتاميثرين). كما أظهرت آليات أنماط المقاومة تردد تطفير عال لـ V1016I وF1534C وإفراط في ، التعبير لـ CCEae3a، *GSTe2* 450 والجينات الأربعة لستوكروم (P، *014614*، *CYP9J23*، *CYP6M11*، *CYP6BB2* ستشكل ). هذه النتائج قاعدة لفهم أعمق لمستويات مقاومة المبيدات الحشرية وآلياتها لبعوض الزاعجة المصرية وسيستخدم لتحسين استراتيجيات مكافحة ناقلات الأمراض في بجزر غوادولوب وسان مارتن.

Translated from English version into Arabic by Halima, through

TRANSLATORS WITHOUT BORDERS

## 瓜德罗普岛和圣马丁岛（法属西印度群岛）埃及伊蚊对溴氰菊酯，马拉硫磷和硫甲双磷的抗性水平及相关机制

Daniella Goindin, Christelle Delannay, Andric Gelasse, Cédric Ramdini, Joël Gustave, Anubis Vega-Rua, Florence Fouque

**摘要**

**引言：**在瓜德罗普岛和圣马丁岛，埃及伊蚊（*Aedes aegypti*）是唯一公认的登革热、基孔肯亚病和 Zika 病毒的传播媒介。40 年来，当地将马拉硫磷作为成蚊杀虫剂，而硫甲双磷作为幼虫杀虫剂使用。自 21 世纪初，欧盟禁止使用这两种杀虫剂后，溴氰菊酯和苏云金杆菌（*Bacillus thuringiensis* var. *israelensis*）就成为瓜德罗普岛的成蚊杀虫剂和幼虫杀虫剂。为了改善瓜德罗普岛和圣马丁岛的媒介控制的管理活动，我们调查了埃及伊蚊对溴氰菊酯、马拉硫磷和硫甲双磷的抗性水平及相关机制。

**方法：**从瓜德罗普岛和圣马丁岛 6 个不同地区采集埃及伊蚊。按照世界卫生组织的建议，采用蚊幼虫测定其对马拉硫磷和硫甲双磷的敏感性，成蚊对溴氰菊酯的敏感性。通过测定击倒抗性(*Kdr*)基因型 V1016I 和 F1534C 突变及解毒机制相关的 8 个酶的表达水平，并且与博拉博拉岛敏感株进行对比研究。

**结果：**抗性倍数($RR_{50}$)结果表明，幼虫对硫甲双磷的抗性较高（8.9-33.1），对马拉硫磷的抗性相对较低（1.7-4.4）。成蚊（雌）对溴氰菊酯表现出中度抗性（8.0-28.1）。对成蚊抗性分子检测结果显示，抗性等位基因 V1016I 和 F1534C 频率较高（V1016I：85%-96%，F1534C：90%-98%）。除此之外，谷胱甘肽 s-转移酶基因、*GSTe2*、羧酸酯酶（*CCEae3a*）、细胞色素基因（*014614*、*CYP6BB*2、*CYP6M11* 和 *CYP9J23*）均过表达。
**结论：**瓜德罗普岛和圣马丁岛的埃及伊蚊对有机磷酸酯类（马拉硫磷和硫甲双磷）和拟除虫菊酯类（溴氰菊酯）表现出多重抗性。抗性模式相关机制显示存在较高频率的 F1534C 和 V1016I *Kdr* 突变，此外，*CCEae3a*、*GSTe2*，及 4 个细胞色素 P450 基因（*014614*、*CYP9J23*、*CYP6M11*、*CYP6BB2*）均过表达。这些结果将成为该地区埃及伊蚊群体抗性水平的基线资料，并且将有助于改善瓜德罗普岛和圣马丁岛的媒介控制策略。

Translated from English version into Chinese by Xin-Yu Feng, edited by Pin Yang

CHINESE CENTER FOR DISEASE CONTROL AND PREVENTION
NATIONAL INSTITUTE OF PARASITIC DISEASES

**Niveaux de résistance des insecticides à la deltaméthrine, au malathion et au téméphos et mécanismes associés dans les moustiques *Aedes aegypti* de Guadeloupe et des îles Saint-Martin (Antilles françaises)**

Daniella Goindin, Christelle Delannay, Andric Gelasse, Cédric Ramdini, Joël Gustave, Anubis Vega-Rua, Florence Fouque

**Résumé**
**Contexte**: En Guadeloupe et aux îles Saint-Martin, les moustiques *Aedes aegypti* sont les seuls vecteurs reconnus des virus de la dengue, du chikungunya et du Zika. Pendant environ 40 ans, contre les moustiques, le malathion était utilisé comme un produit adulticide et le téméphos larvicide. Depuis que l'Union européenne a interdit l'utilisation de ces deux molécules insecticides dans les années 2000, le deltaméthrine et le *Bacillus thuringiensis* var. *israelensis* sont respectivement les adulticides et larvicides qui restent utilisés en Guadeloupe. Afin d'améliorer la gestion de la lutte antivectiorielle en Guadeloupe et à Saint-Martin, nous avons étudié la résistance de *Ae. aegypti* aux mécanismes associés avec la deltaméthrine, le malathion et le téméphos.
**Méthodes:** des moustiques *Ae. aegypti* ont été récupéré dans six différentes localités de Guadeloupe et de Saint-Martin. Pour les essais biologiques de malathion et de téméphos, des larves ont été utilisées et pour la deltaméthrine, des adultes, conformément aux recommandations de l'Organisation mondiale de la santé. Le génotypage de la résistance « Knockdown » (*Kdr*) pour les mutations V1016I et F1534C et les niveaux d'expression de huit enzymes impliquées dans des mécanismes de détoxication ont été examinés et comparés avec la référence sensible de la souche Bora Bora.
**Résultats:** Les ratios de résistance ($RR_{50}$) calculés pour les larves de *Æ. aegypti*montrent de hauts niveaux de résistance au téméphos (ratios de 8,9 à 33,1) et des niveaux faibles de résistance au malathion (de 1,7 à 4,4). Les femelles adultes ont démontré des niveaux de résistance modérés à la deltaméthrine concernant le temps nécessaire pour toucher 50 % des individus, variant de 8,0 à 28,1. Des recherches moléculaires sur des moustiques adultes ont montré de hautes fréquences d'allèles résistants pour V1016I et F1534C (respectivement de 85% à 96% et de 90% à 98%), ainsi qu'une surexpression du gène glutathion S-transférase, *GSTe2*, de la carboxylestérase *CCEae3a* et des gènes cytochrome *014614*, *CYP6BB*2, *CYP6M11*, et *CYP9J23*.
**Conclusions:** les populations de *Ae. aegypti* de Guadeloupe et Saint-Martin affichent une résistance multiple aux organophosphates (téméphos et malathion) et aux pyréthrinoïdes (deltaméthrine). Les mécanismes associés à ces modèles de résistance révèlent de fortes fréquences de mutations *Kdr* de F1534C et V1016I et une surexpression de *CCEae3a*, *GSTe2* et de quatre gènes cytochromes P450 (*014614*, *CYP9J23*, *CYP6M11*, *CYP6BB2*). Ces résultats serviront de base pour une meilleure compréhension des niveaux de résistance aux insecticides des populations de *Ae. aegypti* et des mécanismes associés et seront utilisées pour améliorer les stratégies de lutte antivectiorielle en Guadeloupe et à Saint-Martin.

Translated from English version into French by Kevin Fernandez, through

**Уровни резистентности к инсектицидам делтаметрин, малатион и темефос комаров Aedes aegypti на островах Гваделупа и Сен-Мартен (Французская Вест- Индия) и связанные с ними механизмы**

Даниэлла Гоиндин (Daniella Goindin), Кристел Деланни (Christelle Delannay), Эндрик Желасс (Andric Gelasse), Седрик Рамдини (Cédric Ramdini), Джоэл Густав (Joël Gustave), Анубис Вега-Руа (Anubis Vega-Rua), Флоренс Фуке (Florence Fouque)

**Аннотация**
**Краткое описание.** На островах Гваделупа и Сен-Мартен комары Aedes aegypti являются единственными выявленными переносчиками вирусов лихорадок денге, чикунгунья и Зика. На протяжении 40 лет малатион применялся для борьбы со взрослыми насекомыми и темефос для уничтожения личинок. С тех пор как Европейский Союз запретил использование этих двух инсектицидов в первом десятилетии 21-го века, на Гваделупе в качестве препаратов, воздействующих на взрослые особи и личинки соответственно, применяются только делтаметрин и Bacillus thuringiensis var. Israelensis. Мы исследовали резистентность Ae. aegypti к делтаметрину, малатиону и темефосу, а также связанные с этим механизмы, с целью улучшения борьбы с переносчиками вирусов на Гваделупе и Сен-Мартене.
**Методы.** Комары Ae. aegypti были собраны в шести разных местах на Гваделупе и Сен-Мартене. Согласно рекомендациям Всемирной организации здравоохранения личинки использовались в биопробах на малатион и темефос, и взрослые особи в биопробах на делтаметрин. Было проведено генотипирование для выявления резистентности к нокдаун-эффекту (*Kdr*) мутаций V1016I и F1534C, и были проанализированы уровни экспрессии восьми ферментов, вовлеченных в механизмы детоксикации, в сравнении с чувствительным эталонным штаммом Бора-Бора.
**Результаты.** Уровни резистентности ($RR_{50}$), вычисленные для личинок Ae. Aegypti, показали высокую сопротивляемость к темефосу (от 8,9 до 33,1-кратного) и низкую сопротивляемость к малатиону (от 1,7 до 4,4-кратного). У взрослых самок были обнаружены умеренные уровни сопротивляемости к делтаметрину, варьирующиеся от 8,0 до 28,1-кратного, в отношении ко времени, требуемому для воздействия на 50% особей. Молекулярное исследование взрослых комаров показало высокие резистентные частоты аллеля у V1016I и F1534C (от 85% до 96% и от 90% до 98% соответственно), а также сверхэкспрессию гена глутатион-S-трансфераз GSTe2, карбоксилэекстераза CCEae3a и генов цитохром 014614, CYP6BB2, CYP6M11 и CYP9J23.
**Заключение.** Популяции Ae. aegypti на Гваделупе и Сен-Мартене демонстрируют множественную устойчивость к органофосфатам (темефос и малатион) и пиретроидам (делтаметрин). Механизмы, связанные с такими закономерностями резистентности, показывают высокие частоты Kdr мутаций F1534C и V1016I, сверхэкспрессию CCEae3a, GSTe2 и четырех генов (014614, CYP9J23, CYP6M11, CYP6BB2) цитохрома P450. Полученные результаты послужат основой для более глубокого понимания уровней резистентности к инсектицидам популяций Ae. aegypti и связанных с ними механизмов, и они будут использованы для улучшения способов борьбы с переносчиками вирусов на островах Гваделупа и Сен-Мартен.

Translated from English version into Russian by Natalia Potashnik

**Niveles de resistencia a los insecticidas deltametrina, malatión y temefos y mecanismos asociados en los mosquitos *Aedes aegypti* de las islas de Guadalupe y San Martín (Antillas francesas)**

Daniella Goindin, Christelle Delannay, Andric Gelasse, Cédric Ramdini, Joël Gustave, Anubis Vega-Rua, Florence Fouque

**Resumen**

**Antecedentes**: En las islas de Guadalupe y San Martín, los mosquitos *Aedes aegypti* son los únicos vectores reconocidos para los virus dengue, chikungunya y zika. Durante alrededor de 40 años, se utilizó malatión como adulticida y temefos como larvicida de mosquitos. Desde que la Unión Europea prohibió el uso de estas dos moléculas insecticidas en la primera década del siglo 21, el adulticida y larvicida que quedaron fueron deltametrina y *Bacillus thuringiensis* var. *israelensis* respectivamente que se utilizan en Guadalupe. Para mejorar el manejo de las actividades de control del vector en Guadalupe y San Martin, investigamos la resistencia de *Ae. aegypti* y los mecanismos asociados con deltametrina, malatión y temefos.

**Métodos:** Se recolectaron mosquitos *Ae. aegypti* en seis localidades diferentes en Guadalupe y San Martín. Se utilizaron larvas para bioensayos con malatión y temefos y mosquitos adultos para ensayos con deltametrina, siguiendo las recomendaciones de la Organización Mundial de la Salud. La genotpificación de la resistencia derribante (*Kdr*) (*Knockdown resistance* por su nombre en inglés) para las mutaciones V1016I y F1534C y los niveles de expresión de ocho enzimas relacionadas con los mecanismos de detoxificación se examinaron en comparación con la cepa de referencia susceptible Bora Bora.

**Resultados:** Índices de resistencia ($RR_{50}$) calculados para larvas de *Ae. aegypti* mostraron niveles de resistencia altos a temefos (de 8,9 a 33,1-veces) y bajos niveles de resistencia a malatión (de 1,7 a 4,4-veces). Las hembras adultas mostraron niveles de resistencia moderada a deltametrina en relación al tiempo necesario para afectar a 50% de los individuos, con una variación de 8,0 a 28,1-veces. Investigaciones moleculares en mosquitos adultos mostraron una frecuencia alta de alelos resistente para V1016I y F1534C (de 85% a 96% y de 90% a 98%, respectivamente), así como una sobre expresión de del gen de la glutatión S-transferasa, *GSTe2*, la carboxilesterasa *CCEae3a*, y los genes de los citocromos *014614*, *CYP6BB*2, *CYP6M11*, y *CYP9J23*.

**Conclusiones:** Las poblaciones de *Ae. aegypti* en Guadalupe y San Martín presentan resistencia múltiple a organofosfatos (temefos y malatión), y piretroides (deltametrina). Los mecanismos asociados con estos patrones de resistencia muestran grandes frecuencias de mutaciones F1534C y V1016I *Kdr* y una sobre expresión de *CCEae3a*, *GSTe2*, y cuatro genes del citocromo P450 (*014614*, *CYP9J23*, *CYP6M11*, *CYP6BB2*). Estos resultados constituirán el fundamento para una comprensión más profunda de los niveles de resistencia a insecticidas de las poblaciones de *Ae. aegypti* y serán utilizados para mejorar las estrategias de control del vector en Guadalupe y San Martín.

Translated from English version into Spanish by Claudia Guiraldes, through

TRANSLATORS
WITHOUT BORDERS
