# Supplementary material for: Levels of insecticide resistance to deltamethrin, malathion, and temephos, and associated mechanisms in Aedes aegypti mosquitoes from the Guadeloupe and Saint Martin islands (French West Indies)
Source: Infect Dis Poverty. 2017 Feb 10;6:38. doi: 10.1186/s40249-017-0254-x (PMC5303256; doi:10.1186/s40249-017-0254-x)
Supplement: Additional file 2: — Set of primers used for the expression and amplification of detoxification enzymes q-PCR. (DOCX 14 kb) [file 40249_2017_254_MOESM2_ESM.docx]

**Additional file 2.** Set of primers used for the expression and the amplification of detoxification enzymes qRT-PCR.

| **Sequence (5’ to 3’)** | **Forward/Reverse** | **nt position (transcript)** |
| --- | --- | --- |
| CTATTTCGGAGTCCTAGTGGCC | AeCYP9J28_f | 18 - 39 |
| CTTTGACTCCTCGGTACTTGTCG | AeCYP9J28_r | 192 - 214 |
| AGAATCCACGAAGCGATGAG | AeCYP9J23_f | 1106 - 1087 |
| CTATCCAGGGCGGCAATG | AeCYP9J23_r | 1191 - 1208 |
| AGGTTAAGCAGGAGAGTG | AeCYP6M11_f | 491 - 508 |
| CCTTAGGCATAGTGTTCATC | AeCYP6M11_r | 669 - 688 |
| CTGAAGGGAACCGTCAAGCAA | AeRpL8_f | 235 - 255 |
| TCGGCGGCAATGAACAACT | AeRpL8_r | 335 - 353 |
| GTTGGAGATGAACTCGGACCTG | AeRpS7_f | 277 - 298 |
| GCCTTCTTGCTGTTGAACTCG | AeRpS7_r | 343 - 363 |
| AGTTCAAGGGCCGAGGATTG | AeCYP6BB2_f | 197 - 216 |
| CGGATCCACGAAAATTCCGC | AeCYP6BB2_r | 320 - 339 |
| TCTAAGAAACCCGAATATGACG | AeCCEAE3A_f | 927 - 948 |
| TTGAGGAGGCACGAACAG | AeCCEAE3A_r | 1039 - 1056 |
| GCGGATTTCAGTTGCGTTTCG | AeGSTE2_f | 472 - 492 |
| ACCTGCTCGGCTCCACTTC | AeGSTE2_r | 602 - 620 |
| TGGCTCCAGTCGAGGTAGAA | AeCCEAE6A_f | 1528 - 1547 |
| AAGTTGGTCCACATGCGACA | AeCCEAE6A_r | 1584 - 1603 |
| GGAAGCGTTGAGCATGTGTG | AAEL014614_f | 1616 - 1635 |
| AACTGTGAAACCGTGGGGTC | AAEL014614_r | 1717 - 1736 |
| TCCGGTTCCGTCTGGTATCT | AAEL005950_f | 2602 - 2621 |
| GTGTGTGTAACGGCTCCAGA | AAEL005950_r | 2768 - 2787 |
| AGTCCTGGAAGTTCTGCACG | AAEL007808_f | 711 - 730 |
| AAGGCGACTTTCCGACGAAT | AAEL007808_r | 823 - 842 |
